# COVID-19 and ethics in the ICU

**DOI:** 10.1186/s13054-020-03250-5

**Published:** 2020-08-25

**Authors:** Sarah E. Nelson

**Affiliations:** Departments of Neurology and Anesthesiology & Critical Care Medicine, Johns Hopkins University, 600 N Wolfe Street, Phipps 455, Baltimore, MD 21287 USA

The COVID-19 pandemic has brought into focus many medical ethics questions; several have burdened intensive care unit physicians in particular (Fig. 1). The aim of this article is to provide a frank yet thoughtful discussion of the many facets of these ethical dilemmas.

**Fig. 1:**
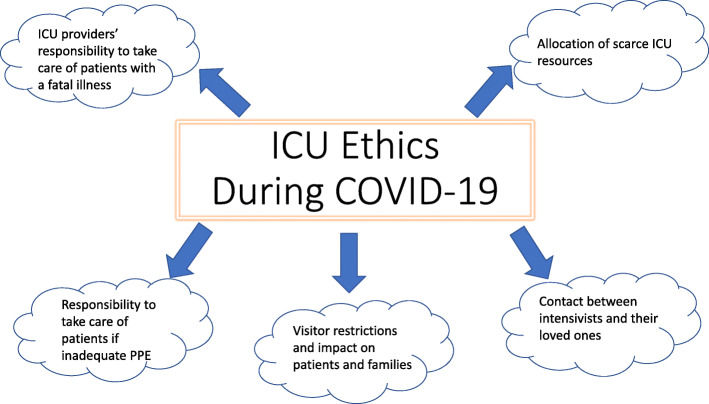
Medical ethics issues encountered by intensivists during the COVID-19 pandemic

We intensivists potentially put ourselves at risk each time we report to work. This is scary and unsettling. Hundreds of years ago, physicians had no professional or ethical obligation to take care of sick patients during disease outbreaks, some purposely fleeing from plague-ridden areas [1]. However, times have changed. Most medical students now recite the Hippocratic Oath, which states that: “I will apply, for the benefit of the sick, all measures [that] are required,” though nowhere does it state that physicians must work in settings that could put their own health at risk [2]. Following the events of September 11, 2001, the American Medical Association (AMA) reaffirmed their stance that “it is a responsibility of health professionals to continue caring for patients even if doing so presents some danger to them” [1]. This includes an “obligation to provide urgent medical care during disasters … even in the face of greater than usual risks to physicians’ own safety, health, or life.” Given the large scope of the pandemic and the deadliness of SARS-CoV-2, these statements may not adequately address this ethical quandary. For instance, it is not entirely clear how much of a hazard is actually acceptable. And risk to healthcare workers is real: > 3300 were infected in China as of early March 2020 as were 20% of healthcare workers in Italy [3]; hundreds of these workers around the world have died since the pandemic began [4]. Healthcare providers may feel they did not sign up for their jobs knowing that they may need to sacrifice themselves for others [5]. In addition, hospitals have periodically suffered from shortages of personal protective equipment (PPE). Is it ethical to ask intensivists and other healthcare providers to counter this contagious threat with inadequate battle gear?

One of the most upsetting issues of the pandemic is the limited number of critical care resources, including ventilators and intensive care unit beds, available to COVID-19 patients and other critically ill patients. In countries such as Italy, healthcare providers have had to make difficult decisions about who is provided a ventilator and who is not and thus forced to dictate life and death [6], and other countries have prepared to face similar difficult decisions. In what almost seems a foreshadowing, distribution of limited resources was explored recently by a Johns Hopkins intensivist and her team. Participants in the study felt that, in times of crisis, short- and long-term outcomes should be primarily considered (versus a lottery system or first come, first served) in deciding who should be prioritized to receive scarce resources [7]. The same research group then generated a framework based on the 2 major ethical considerations they had identified: short-term survival, with the support of all available resources, and long-term survival, with consideration of comorbidities [8].

Specific to the COVID-19 pandemic, 4 ethical guiding principles to consider when resources are limited have been noted: (1) maximizing benefits of scarce resources, (2) treating all people equally, (3) preferentially selecting those with instrumental value, and (4) prioritizing patients who are worst off [9]. Based on these principles, 6 recommendations have been made for the current outbreak: maximizing benefits including using scarce resources responsibly and saving more lives/years of life, prioritizing COVID-19 resources (i.e., PPE, vaccines) to healthcare workers, invoking equality using random allocation or lottery to distribute resources to those with similar prognoses, thoughtful consideration of resource allocation (e.g., prioritizing older patients, among the most affected by SARS-CoV-2, to receive a vaccine), prioritizing those who have participated in COVID-19-related research, and providing equal resources to those with COVID-19 and those with other medical conditions [9].

In response to the pandemic, The Johns Hopkins Hospital convened a Scare Resource Taskforce to reach a consensus about available resources in Maryland and triggers for initiating a framework requiring allocation of scarce resources. Other US states have formally approved a scare resource allocation protocol, and individual medical centers have been forced to put together such polices or released recommendations regarding issues to consider when designing them [10, 11]. One of the most severely afflicted epicenters in the world, New York State has instituted legal immunity for physicians forced to make these difficult triage decisions. Given the increasing number of COVID-19 cases, the potential need to triage critical care resources has deeply affected physicians [12].

Further, visitor restrictions in hospitals have led to emotionally painful experiences. Visitors can augment patient histories and provide sources of comfort to patients. Unfortunately, by increasing the number of people in a hospital at one time, hospital visitors can potentially hamper social distancing and contribute to increased spread of SARS-CoV-2. As a result, many hospitals have closed their doors to visitors except under special circumstances. In one of the more heartbreaking aspects of this crisis, many patients have therefore died alone and without loved ones at their bedside [13]. Inpatients admitted for other reasons have also been affected and unable to communicate with their families in person [14].

Another ethical dilemma exists at home. What level of interaction after a day at work is safe between healthcare providers caring for COVID-19 patients and their families? While essential workers wish to sustain their personal relationships, their interactions with other people after working in the hospital could possibly increase spread of SARS-CoV-2 and cause additional suffering. Many physicians have struggled with this quandary [15]. In a similar vein, large family gatherings including weddings and funerals have been impacted and thus forced to downsize, which has also created great distress.

Several ethical dilemmas associated with the COVID-19 pandemic affect intensive care physicians. No one yet knows the full extent of the psychological injury that these issues have caused, but unfortunately it will likely be significant.

## Data Availability

Upon request.
